# Arthritis, osteomyelitis, septicemia and meningitis caused by *Klebsiella *in a low-birth-weight newborn: a case report

**DOI:** 10.1186/1752-1947-5-241

**Published:** 2011-06-27

**Authors:** Ziaaedin Ghorashi, Nariman Nezami, Hamideh Hoseinpour-feizi, Sona Ghorashi, Jafar Sadegh Tabrizi

**Affiliations:** 1 Department of Pediatrics, Tabriz University (Medical Sciences), Tabriz, Iran; 2 Drug Applied Research Center, Tabriz University (Medical Sciences), Tabriz, Iran; 3 Young Researchers Club, Tabriz Branch, Islamic Azad University, Tabriz, Iran; 4 Faculty of Health and Nutrition, Tabriz University of Medical Sciences, Tabriz, Iran

## Abstract

**Introduction:**

*Klebsiella pneumoniae *is in most cases a hospital-acquired infection and presents as pneumonia, septicemia and meningitis in patients with some predisposing factors, including prematurity, intravenous catheter, history of antibiotic therapy and intravenous nutrients.

**Case presentation:**

A low-birth-weight, 33-day-old Caucasian girl with respiratory distress syndrome was admitted to our hospital. She developed septicemia, meningitis, polyarticular arthritis and osteomyelitis by nosocomial *K. pneumoniae *which was resistant to most antibiotics except ciprofloxacin. She was therefore treated with ciprofloxacin and co-trimoxazole for eight weeks. After completion of the treatment course, she completely improved with excellent weight gain and without any adverse effects during three years of follow-up.

**Conclusion:**

In the resistant strain of *K. pneumoniae*, ciprofloxacin could be considered as a therapeutic option with the prospect of a good outcome, even in neonates and infants.

## Introduction

Pneumonia is a type of infection that is most commonly caused outside the hospital by *Klebsiella pneumoniae *[1]. Mostly, *K. pneumoniae *is recognized as a hospital-acquired infection presenting as pneumonia, septicemia and meningitis in patients with some predisposing factors (including prematurity, intravenous catheter, history of antibiotic therapy and intravenous nutrients) [2,3]. In the rare patients with underlying conditions among newborns and older adults, *K. pneumoniae *may result in arthritis and osteomyelitis. All *Klebsiella *subtypes are resistant to ampicillin, especially multi-drug-resistant (MDR) subtypes which are resistant to the majority of antibiotics, except fourth-generation cephalosporins and carbapenems. Previously, patients with MDR subtype infections usually received first-generation cephalsporins and aminoglycosides.

## Case presentation

A 33-day-old Caucasian girl was brought to the Tabriz Children's Hospital with poor breastfeeding, recurrent vomiting and anorexia. She was admitted with a primary diagnosis of septicemia.

She was born from a mother with pre-eclampsia through normal vaginal delivery at the 34th week of gestation, with a birth weight of 1670 g. Her Apgar scores at one and five minutes were five and six, respectively. During delivery and before admission to the Tabriz Children's Hospital, she had been hospitalized in the Talegani neonatal intensive care unit for prematurity, septicemia, respiratory distress syndrome and gastrointestinal bleeding. In Talegani Hospital, she had received antibiotic therapy, including ampicillin and gentamicin, then her medication was changed to cefotaxime and vancomycin, and finally her treatment continued with intravenous immunoglobulin, imipenem and ceftazidime.

Her physical examination revealed that she was pale, cachectic, anorexic and hypotonic, and her Moro and sucking reflexes were weak. She also exhibited grunting and had substernal and intercostal retraction. The patient's body weight, height and head circumference were 1700 g, 43 cm and 31 cm, respectively. Her vital signs, including pulse rate, respiratory rate, body temperature, O_2 _saturation under the oxygen hood and without using the oxygen hood were 158/min, 48/min, 38.6°C, 95% and 89%, respectively.

**Figure 1:**
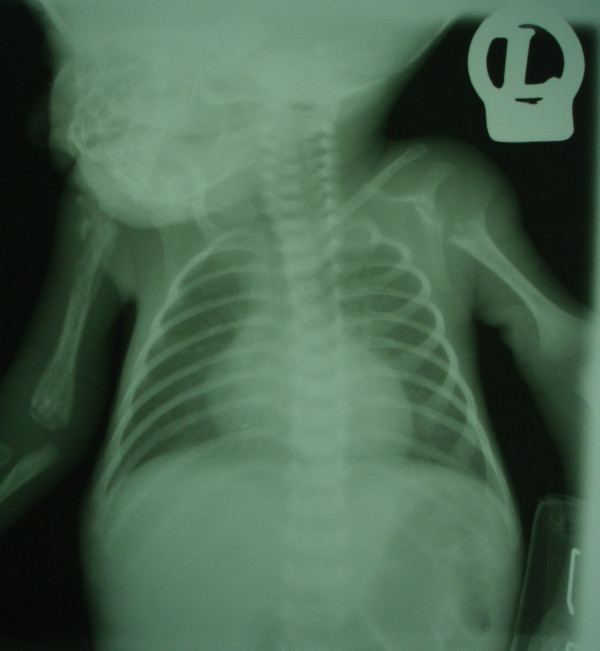
**Chest X-ray obtained at the time of admission**. Bilateral osteomyelitis in the humerus presented as osteolysis and involucrum. Also, there is sequestration because of osteonecrosis and a pathologic fracture in the proximal part of the humerus. Dislocation of right glenohumeral joint brings up the arthritis diagnosis. There is complete osteolysis in proximal metaphysis in the left humerus and arthritis in the left glenohumeral joint. The heart, lung and pleural space have a normal appearance, while the thymus is atrophic.

Her cerebrospinal fluid (CSF) was purulent, and CSF analysis showed 520 mg/dL protein; 16 mg/dL glucose; many white blood cells (WBCs), with 85% polymorphonuclear cells and 15% lymphocytes; and 25 red blood cells (RBCs)/mm^3^. The results of the other laboratory tests are shown in Table 1. In the CSF and blood culture, *K. pneumonia *was resistant to most of the antibiotics and sensitive only to ciprofloxacin and co-trimoxazole. Her chest X-ray showed bilateral humeral osteomyelitis and bilateral glenohumeral joint arthritis (Figure 1).

**Table 1:** Laboratory tests and results on admission^a^

Complete blood cell count		Biochemical analysis		Arterial blood gas	
WBC count (cells/μL)	6500	FBG (mg/dL)	42	pH	7.29
Hb, g/Dl	7.8	Cr (mg/dL)	0.5	HCO_3_^- ^(mmol/L)	20
Platelets, *n*/μL	773 × 10^3^	BUN (mg/dL)	24	PCO_2 _(mmHg)	43
PMN cells, %	51				
Eosinophils, %	1%	Electrolytes		Other	
		
Lymphocytes, %	24%	Na (mEa/L)	136	CRP	2+
Monocytes, %	2%	K (mEa/L)	45	Blood group	A^+^
Band cells, %	21%	Ca (mg/dL)	10.8		

^a^WBC, white blood cells; Hb, hemoglobin; PMN, polymorphonuclear cells; CRP, C-reactive protein; Na, sodium; K, potassium; BUN, blood urea nitrogen; FPG, fasting plasma glucose; Ca, calcium.

**Figure 2:**
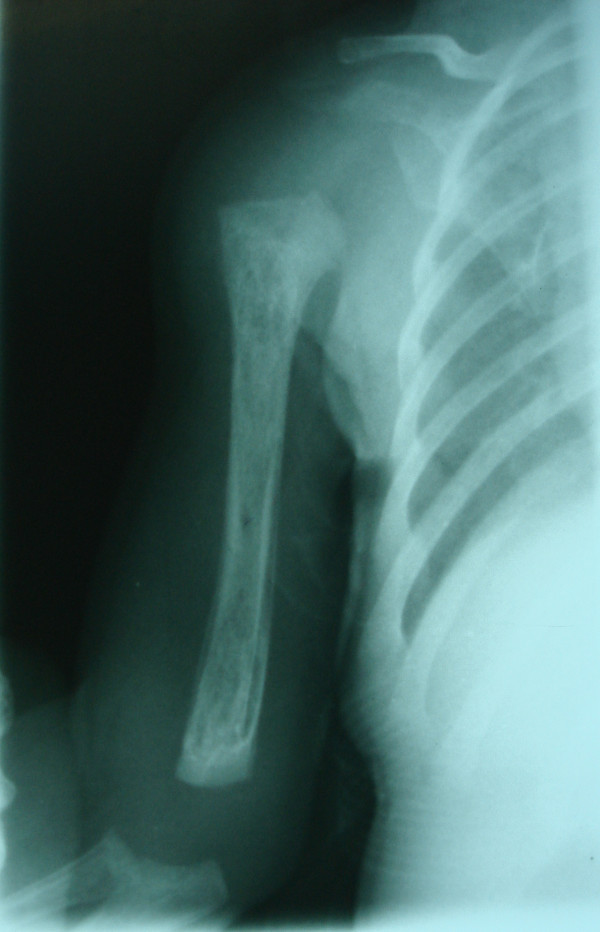
**Chest X-ray obtained on the seventh day of oral anti-biotic therapy**. The lytic lesions in the proximal and distal metaphysis in the right humerus are shown.

**Figure 3:**
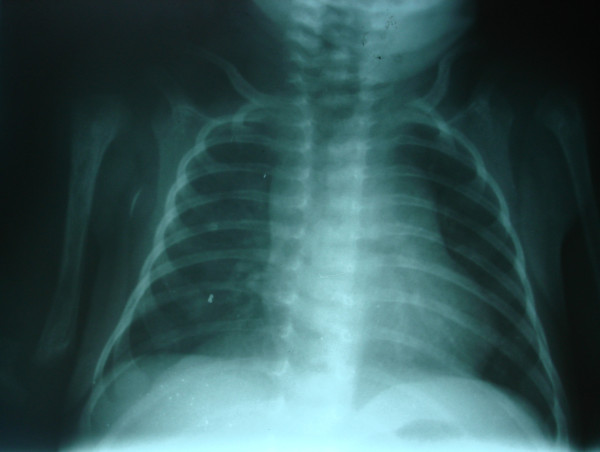
**Chest X-ray showing complete resolution of symptoms at the end of anti-biotic therapy**.

On the basis of the paraclinical evidence, diagnoses of *K. pneumoniae *septicemia, meningitis, arthritis and osteomyelitis were made, and a treatment protocol with a combination of intravenous ciprofloxacin and co-trimoxazole antibiotics was started (for 28 days). At the end of the intravenous treatment period, she weighed 2420 g, and her CSF analysis and culture were within normal range. Afterward, she was discharged with oral ciprofloxacin, co-trimoxazole and rifampicin for another 28-day period. Figures 2 and 3 show her chest X-rays obtained on the seventh and 28th days of oral antibiotic therapy, respectively. At the end of 28 days of oral antibiotic therapy (when the patient was 88 days old), her weight had reached 4250 g and normal glenohumeral joints and humerus bones were shown on her chest X-ray. During three years of follow-up, she had normal developmental milestones and was not readmitted to the hospital.

## Discussion

Prolonged hospital stay, decreased gestational age, prolonged use of broad-spectrum antibiotics and inadequacy of some basic facilities and staffing carry the risk of introduction of resistant hospital pathogens [4-6]. In the present case, all of the above-mentioned factors, combined with prematurity, predisposed the neonate to a higher risk of contracting nosocomial *K. pneumoniae *arthritis, osteomyelitis, septicemia and meningitis, although the common cause of osteoarthritis is Gram-positive cocci [7].

Adeyemo *et al*. [8] reported an outbreak of bone infections associated with neonatal septicemia by *K. pneumoniae *in 12 neonates over a six month period at the Special Care Baby Unit, University College Hospital, Ibadan, Nigeria. All patients had septic arthritis, 10 of them had osteomyelitis and 50% had multiple-joint involvement.

Hospital-acquired *K. pneumoniae *has been reported to be resistant to multiple antibiotics [8,9]. In addition, Ghahramani and Nahaie [10] showed that *K. pneumoniae *is the most common cause of septicemia in the neonatal ward of the Tabriz Al-Zahra Gynecology and Obstetrics Referral Hospital in Tabriz, Iran.

The parenteral third-generation cephalosporins appear to be a major therapeutic advance in the treatment of *K. pneumoniae *[11], but reports of highly resistant strains that produce plasmid-mediated, extended-spectrum β-lactamases influenced therapeutic outcomes again [12]. Evidence revealed that *K. pneumoniae *infection, especially the nosocomial type, is resistant to the majority of antibiotics except for ciprofloxacin and ofloxacin [13]. In the present case report, the isolated *K. pneumoniae *was resistant to most of the antibiotics except ciprofloxacin and co-trimoxazole. Therefore, these two antibiotics were used in the treatment protocol. Because of the quinolone cartilage toxicity potential in experimental juvenile animal models, the use of ciprofloxacin among children has been restricted [13,14]. However, recent data from Bayer's ciprofloxacin clinical trials database indicate that the role of fluoroquinolones in the treatment of certain serious infections in children does not appear to be compromised by safety concerns when used appropriately [15]. In such cases, when a micro-organism is resistant to all antibiotics except ciprofloxacin, a dosage of 15 mg/kg/day to 30 mg/kg/day is advised in neonates [16].

After completion of the treatment course, our patient completely improved and achieved normal developmental milestones and weight gain, without adverse effects or hospital readmission during three years of follow-up.

## Conclusion

In neonates with delivery problems, prematurity, low birth weight and prolonged hospital admission, nosocomial *K. pneumoniae *should be considered in the differential diagnosis of septicemia, arthritis, osteomyelitis and meningitis. Considering the multi-drug resistance of nosocomial *K. pneumoniae *and sensitivity to quinolones, ciprofloxacin, when used appropriately, should be considered a therapeutic option with good outcomes in patients with serious infections with resistant strains of *K. pneumoniae*, even in neonates and infants.

## Abbreviations

CSF: cerebrospinal fluid; MDR: multi-drug-resistant.

## Consent

Written informed consent was obtained from the patient's next-of-kin for publication of this case report and any accompanying images. A copy of the written consent is available for review by the Editor-in-Chief of this journal.

## Competing interests

The authors declare that they have no competing interests.

## Authors' contributions

ZG, HH and SG collected the patient data and participated in the patient's hospitalization and treatment process. NN was a major contributor in writing the manuscript. JST helped to revise and edit the manuscript. All authors read and approved the final manuscript.
